# Micronutrient malnutrition in geriatric patients

**DOI:** 10.1016/j.jnha.2024.100174

**Published:** 2024-02-02

**Authors:** Dorothee Volkert

**Affiliations:** Institute for Biomedicine of Aging, Friedrich-Alexander-Universität Erlangen-Nürnberg, Germany

With regard to malnutrition in older persons, over the last few decades, clearly protein-energy malnutrition has been at the center of our interest [1], and micronutrients have somewhat escaped our attention. In this issue, Yilmaz and colleagues now have addressed this somewhat neglected but highly relevant topic of micronutrient malnutrition in geriatric patients [2]. In a sample of 156 malnourished patients from a German geriatric acute care hospital unit, serum concentrations of 10 vitamins and 4 trace elements were analysed, and impressively high prevalence rates of values below usual reference ranges are reported [2]. In a multivariate analysis, multiple (>2) micronutrient deficiency was found to be related to previous unintended weight loss, implying an overlap between protein-energy and micronutrient malnutrition.

Already in 1992, vitamin deficiencies were found to be widespread among hospitalised geriatric patients in Germany. That time, for example, low plasma concentrations of vitamin C were observed in 37 % of the whole patient sample and in about half of those who were clinically judged to be undernourished. Furthermore, the clinical diagnosis of undernutrition was associated with a high prevalence of low biochemical values, which even then showed the close connection between general, more obvious malnutrition and the more hidden shortage of micronutrients [3].

The current analyses of Yilmaz et al. [2], however, in addition to being up-to-date, are much more comprehensive than the previous ones, and the results are alarmingly worse: in every patient at least one abnormal micronutrient value was observed, and multiple abnormal values were found in 90 %.

Though, when interpreting these results, some points must be taken into account.

First, it must be emphasised, that all patients under study were malnourished according to MNA or weight loss, most of them were frail and characterised by pronounced physical and mental limitations. Our knowledge of the meaning of serum micronutrient concentrations in this particular patient group is actually very limited. Blood values have very different significance for different nutrients and do not necessarily reflect the overall nutritional status of the body [4]. Atypical laboratory findings are quite common in geriatrics [5].

Further, prevalence figures depend on the reference values used. These are usually derived from young, healthy persons and we know very little about whether they are also appropriate for the specific target group of very old, acutely ill persons. This is reflected in different reference values used for older hospitalised patients by different working group, e.g. in five publications reporting vitamin C deficiency, five different cut-off values were applied to define “deficiency” [2,3,[6], [7], [8]].

Ultimately, clinical relevance of these values is of the utmost importance. Are there any clinical correlates and consequences of such values? Surprisingly, a decade ago, in geriatric patients in Paris, even clinical signs of vitamin deficiency were reported. Eighteen out of 145 consecutive patients (12 %) actually had scurvy symptoms. In all except one of these patients, serum vitamin C levels were markedly reduced [6]. More recently, case reports about scurvy [9] and Wernicke encephalopathy in older persons due to chronic malnutrition [10] were published.

When reading these articles, I was more than surprised and wondering how such extreme deficiencies can occur in the middle of our affluent society. In fact, it is quite conceivable that very old, handicapped people may not be able to obtain and consume sufficient fresh, nutrient-rich food for longer periods of time due to already impaired health before they arrive at the hospital. This consideration, however, has a clear social and societal dimension, as nutritional care of very old persons in our societies obviously seems to be inadequate. Interesting in this regard: in the French study, scurvy symptoms were related to the need for assistance with eating [6]. Also, financial problems may have contributed [11], besides all other potential causes of inadequate nutrition [12].

Regardless, the question about the significance of subclinical inadequacies remains.

It could legitimately be objected that the values are only temporarily reduced due to the acute illness and improve on their own as part of the recovery process or that they might be an expression of the end of life. Unfortunately, most of the available evidence is only of cross-sectional origin and does not give information about causes and consequences and also not about the course of these values. From a physiological point of view, however, all essential nutrients have important body functions – from immune response to wound healing and cognition – and an insufficient supply, which is at least for some of the nutrients reflected in reduced serum concentrations, will sooner or later result in more or less pronounced functional disorders.

It is not acceptable that longer standing and pronounced inadequacies develop in our surroundings, and it is even more unacceptable that these conditions obviously go completely unnoticed in the acute care setting.

Health care professionals should pay attention, keep potential micronutrient deficiencies in mind and thus recognise them early. In patients with a suspected deficiency, biochemical information may help to estimate the dimension of the deficiency and decide on the appropriate supplement dosage. Comprehensive practical guidance on the assessment of micronutrient status and for micronutrient supplementation – albeit with a focus on enteral and parenteral nutrition – is available in a current ESPEN guideline [4].

In high-risk groups, like geriatric patients with general malnutrition (indicated e.g. by a significant weight loss), but also malnourished long-term care residents [13], in addition to other treatment options [14], generous supplementation of micronutrients might be justified despite a lack of scientific evidence. Given the obviously widespread presence of multiple micronutrient inadequacies [2] and very limited risk of harm with amounts in the range of daily intake recommendations, multi-nutrient supplementation of this magnitude seems appropriate.

Above all, however, adequate nutrition should be enabled and ensured in every patient: in hospitals and other institutions by attractive high-quality food containing energy and all essential nutrients in sufficient quantities, but also at home, e.g. by oranising help for shopping or a dental visit, by prescribing individually helpful eating utensils or ordering meals on wheels. Implementing good nutritional care in the everyday lives of older people means prevention of macro- as well as micronutrient malnutrition.

At the same time, well-designed and well-performed studies are required which need to bring the evidence, which amounts of which nutrients lead to clinically relevant benefits at which serum values. Simply correcting abnormal blood values does not yet improve the quality of life of those affected!

## References

[1] Volkert D., Visser M., Corish C.A., Geisler C., de Groot L., Cruz-Jentoft A.J., MaNuEL consortium (2020). Joint action malnutrition in the elderly (MaNuEL) knowledge hub: summary of project findings. Eur Geriatr Med.

[2] Yilmaz K., Wirth R., Daubert D., Pourhassan M. (2024). Prevalence and determinants of micronutrient deficiencies in malnourished older hospitalised patients. J Nutr Health Aging.

[3] Volkert D., Kruse W., Oster P., Schlierf G. (1992). Malnutrition in geriatric patients: diagnostic and prognostic significance of nutritional parameters. Ann Nutr Metab.

[4] Berger M.M., Shenkin A., Schweinlin A., Amrein K., Augsburger M., Biesalski H.-K. (2022). ESPEN micronutrient guideline. Clin Nutr.

[5] Michel J.-P., Beatti B.L., Martin F.C., Walston J.D. (2018).

[6] Raynaud-Simon A., Cohen-Bittan J., Gouronnec A., Pautas E., Senet P., Verny M. (2010). Scurvy in hospitalized elderly patients. J Nutr Health Aging.

[7] Paillaud P., Merlier I., Dupeyron C., Scherman E., Poupon J., Bories P.-N. (2004). Oral candidiasis and nutritional deficiencies in elderly hospitalised patients. Br J Nutr.

[8] Sharma Y., Popescu A., Horwood C., Hakendorf P., Thompson C. (2022). Relationship between vitamin C deficiency and cognitive impairment in older hospitalised patients: a cross-sectional study. Antioxidants.

[9] Callus C.A., Vella S., Ferry P. (2018). Scurvy is back. Nutr Metab Insights.

[10] Shah F.A., Moronta S., Braford M., Fujikawa P.Y., Stocker G. (2021). Wernicke encephalopathy in an elderly patient due to chronic malnutrition from an atypical diet. Cureus.

[11] Rigling M., Schuetz P., Kaegi-Braun N. (2023). Is food insecurity contributing to malnutrition in older adults in Switzerland? A cross-sectional study. Front Nutr.

[12] Volkert D., Kiesswetter E., Cederholm T., Donini L.M., Eglseer D., Norman K. (2019). Development of a model on determinants of malnutrition in aged persons: a MaNuEL project. Gerontol Geriatr Med.

[13] Sanchez M., Courtois-Amiot P., Capdepon A., Neveux N., Gautry J., Dorigny B. (2023). Four-week administration of an energy and protein dense oral nutritional supplement improves micronutrient concentrations but does not completely correct deficiencies in institutionalized malnourished older adults. Front Nutr.

[14] Volkert D., Beck A.M., Cederholm T., Cruz-Jentoft A., Hooper L., Kiesswetter E. (2022). ESPEN practical guideline: clinical nutrition and hydration in geriatrics. Clin Nutr.

